# Capacities for resilience in healthcare; a qualitative study across different healthcare contexts

**DOI:** 10.1186/s12913-022-07887-6

**Published:** 2022-04-10

**Authors:** Hilda Bø Lyng, Carl Macrae, Veslemøy Guise, Cecilie Haraldseid-Driftland, Birte Fagerdal, Lene Schibevaag, Siri Wiig

**Affiliations:** 1 SHARE - Centre for Resilience in Healthcare, Faculty of Health Sciences, University of Stavanger, N-4036 Stavanger, Norway; 2 Nottingham University Business School, University of Nottingham, Nottingham, UK

**Keywords:** Resilience in healthcare, Organizational resilience, Capacities, Operationalization

## Abstract

**Background:**

Despite an emerging consensus on the importance of resilience as a framework for understanding the healthcare system, the operationalization of resilience in healthcare has become an area of continuous discussion, and especially so when seeking operationalization across different healthcare contexts and healthcare levels. Different indicators for resilience in healthcare have been proposed by different researchers, where some indicators are coincident, some complementary, and some diverging. The overall aim of this article is to contribute to this discussion by synthesizing knowledge and experiences from studies in different healthcare contexts and levels to provide holistic understanding of capacities for resilience in healthcare.

**Methods:**

This study is a part of the first exploratory phase of the Resilience in Healthcare programme. The exploratory phase has focused on screening, synthesising, and validating results from existing empirical projects covering a variety of healthcare settings. We selected the sample from several former and ongoing research projects across different contexts and levels, involving researchers from SHARE, the Centre for Resilience in Healthcare in Norway. From the included projects, 16 researchers participated in semi-structured interviews. The dataset was analysed in accordance with grounded theory.

**Results:**

Ten different capacities for resilience in healthcare emerged from the dataset, presented here according to those with the most identified instances to those with the least: Structure, Learning, Alignment, Coordination, Leadership, Risk awareness, Involvement, Competence, Facilitators and Communication. All resilience capacities are interdependent, so effort should not be directed at achieving success according to improving just a single capacity but rather at being equally aware of the importance and interrelatedness of all the resilience in healthcare capacities.

**Conclusions:**

A conceptual framework where the 10 different resilience capacities are presented in terms of contextualisation and collaboration was developed. The framework provides the understanding that all resilience capacities are associated with contextualization, or collaboration, or both, and thereby contributes to theorization and guidance for tailoring, making operationalization efforts for the identified resilience capacities in knowledge translation. This study therefore contributes with key insight for intervention development which is currently lacking in the literature.

**Supplementary Information:**

The online version contains supplementary material available at 10.1186/s12913-022-07887-6.

## Introduction

Resilience in healthcare provides a theoretical perspective for understanding complex adaptive systems, and its impact in healthcare studies has increased over recent years [1]. The value gained by using the resilience perspective in healthcare studies is diverse [2]. Firstly, a focus on how healthcare practices need to cope, respond, and adapt to stress allows for a more dynamic understanding of healthcare systems. Secondly, use of the resilience concept has allowed for the import of new ideas to the healthcare sector, since resilience as a theoretical perspective stem from domains like resilience engineering (societal safety), social ecology, and psychology. Thirdly, the resilience concept provides a bridge between different interests (like strategies and agendas) across different healthcare levels and contexts, allowing for a more holistic understanding of the healthcare system [3–5].

Despite an emerging consensus on the importance of resilience as a framework for understanding the healthcare system, the operationalization of resilience in healthcare has become an area of continuous discussion, and especially so when seeking operationalization across different healthcare contexts and healthcare levels [4–7]. One traditional way of seeking to operationalize a concept is to develop indicators. An indicator can be defined as “something that shows what a situation is like”, thus describing both quantitative and qualitative factors as suitable for illustrating a situation [8]. Indicator development is however not a straightforward matter, due to the difficulty in operationalizing different resilience concepts, the desire to avoid context specificity, and a lack of empirical investigations to test theoretical frameworks.

Different indicators or characteristics for resilience in healthcare have been proposed by different researchers, describing resilience in terms of measures, features, a philosophy, or as capabilities [9]. The work of identifying leading indicators for resilience is nonetheless important, particularly as it can allow individual organisations to identify and understand strengths and weaknesses and therefore enable the organization to better prepare for and respond to stress and identify opportunities to bypass challenging situations [9, 10].

This study is a part of the first exploratory phase of the Resilience in Healthcare programme, with the overall aim at providing empirical and analytical indicators and different learning tools for Resilience in Healthcare across contextual settings and levels. This is a contribution to the first step, which seeks to investigate and identify cross-contextual resilience capacities for further use in the process of developing cross-contextual learning tools and empirical and analytical indicators [11, 12].

## Background

The majority of research concerning resilience in healthcare has focused on shocks and crises, like pandemics and natural disasters [13]. However, the importance of resilience for everyday healthcare operations, aimed at maintaining quality care, has gained increased attention in recent years [6]. Based on the latter we ground our understanding of resilience in healthcare in the following definition of resilience from Wiig et al., [ [1], p. 6]; “the capacity to adapt to challenges and changes at different system levels, to maintain high quality care”*.* In addition, we acknowledge how Kruk et al. [2], describe that a system’s ability to succeed in situations of chronic stress can also strengthen the system’s ability to manage well in the face of sudden shocks. This means, therefore, that capacities for resilience that are developed in and support everyday practices are closely related to the resilience that may be activated in shocks and crises.

A framework for understanding the importance of adaptive capacity for ensuring resilience in healthcare, named the CARE model, has been provided by Anderson et al. [14]. This framework, with its foundation in Resilience Engineering concepts, illustrates the need for adaptations to narrow or close the gap between work as done and work as imagined. The outcomes of adaptations may end up as both acceptable or unacceptable, which corresponds to the performance variability theory by Hollnagel et al. [15].

In the existing literature on resilience in healthcare, diverse contributions have provided valuable understanding of different components of resilience. In terms of crises, Kruk et al. [[2],] found five features to be important for achieving resilience: awareness, integrative factors (coordination and involvement of different actors), self-regulation, adaptive capabilities, and a diverse category including aspects like effective responses and economic issues. The authors emphasize that these features do not provide resilience in themselves, but also rely on a foundation of leadership, a committed workforce, appropriate infrastructure, and global support. The complex nature of resilience, and the various factors and processes that support it, means that there can be no single indicator for resilience. Rather, a host of interrelated factors needs to be considered to fully understand how an organization can support resilient performance.

Likewise, a recent review of the nature of organizational resilience in crises across multiple sectors identified 10 factors which nurture organizational resilience: Material resources, preparedness and planning, information management, collateral pathways and redundancy, governance processes, leadership practices, organizational culture, human capital, social networks, and collaboration [13].

Contributions from the field of social ecology can also inform our understanding of organizational resilience. Within this research tradition, a tool has been developed to measure organizational resilience in response to emergencies and crisis. Three main factors made up the structure of this tool: Situation awareness, Management of keystone vulnerabilities, and Adaptive capacity [10, 16]. Other conceptions of organizational resilience ground their understanding of resilience in theories of High Reliability Organizations (HRO) [17], viewing organizational resilience as emerging from organisational capacities for adaptation, rapid communication, buffers, and an emphasis on learning and expertise.

In more recent developments based on a resilience engineering tradition, Hollnagel [18–20] focuses on four ‘potentials’ for resilient performance in healthcare: Anticipation, Monitoring, Responding and Learning. The anticipation potential refers to an ability that extends conventional risk assessment, by having an awareness of future events, situational issues, and changes that takes place [18]. Anticipation is therefore important for understanding the consequences of adaptations and relies on situational understanding. Monitoring refers to an ability to understand what to look for so that changes, positive or negative, can be identified. The potential of responding concerns the ability to respond in a good way to challenges and changes and as such also relies on contextual competence. The ability to learn is the final potential, emphasizing the need for learning from both positive and negative experiences and outcomes [18]. These potentials have been used to operationalize resilience both qualitatively and quantitatively in healthcare settings [21, 22] and as input for the Resilience Analysis Grid (RAG) [18]. However, a consensus of indicators for how to operationalize these potentials is yet to be developed across different levels and contexts and new research to seek such understanding has been called for [3, 5].

This summary of previous work illustrates the ambiguity in both the terms used in and the content of different resilience studies. Some researchers use the term ‘indicators’, while others use ‘factors’, ‘features’, or ‘potentials’. These diverse contributions also illustrate differences in what is included as markers of resilience. However, there are also many similarities across the different studies, such as the importance of the concept of adaptive capacity, which is emphasized in most of the attempts at operationalizing resilience. Other factors that reoccur in different studies are issues of leadership and awareness, planning, and anticipation. When seeking operationalization of resilience in healthcare, researchers need to carefully consider what purpose and whom this operationalization is meant to serve. Indicators for organizational resilience across disciplines are traditionally intended for research or policy use [23]. Less consideration has been given to how the actors who are closer to operational work, may use indicators, to support increased reflexivity of resilience for healthcare professionals and managers in healthcare [24].

### Aim

The aim of this study is to clarify and contribute to ongoing debates about the operationalization of resilience. This study is a part of the first exploratory phase of the Resilience in Healthcare (RiH) research programme which will develop collaborative learning tools to support resilience in healthcare for a variety of end users [16, 17]. As such, in this article we aim to synthesise findings from studies from different healthcare contexts and levels to provide holistic understanding of capacities for resilience in healthcare for future use in different learning tools [11, 12]. By investigating previous and ongoing health services research studies, this analysis explores the resilience mechanisms and capacities that need to be considered when translating between theoretical models and practical improvement in resilience in healthcare. This study therefore contributes key insight for intervention development which is currently lacking in the literature [5].

The research question for this study is therefore as follows; What type of capacities for resilience can be identified across different healthcare contexts and levels?

The term capacity can be defined as an actual or potential ability to perform or withstand [25] and is therefore linked to the definition of resilience in healthcare that grounds this study (adapt to challenges and changes [1]) and is thus chosen to describe the findings in this paper.

## Methods

### Research design and sample selection

The exploratory phase of the RiH programme has focused on screening, synthesising, and validating results from existing empirical projects covering a variety of healthcare settings. Results from this exploration phase will form the backdrop of an intervention phase that includes design, implementation, and evaluation of measures to facilitate resilient capacities in healthcare systems [12]. This includes developing actionable collaborative learning tools and principles for supporting resilience in diverse healthcare contexts [11, 12].

In the exploratory phase we have used data from a sample of research projects, from multiple empirical healthcare settings, across all levels of the healthcare system (micro, meso, macro). We selected the sample from several former and ongoing research projects involving researchers from the Centre for Resilience in Healthcare (SHARE) in Norway. For the selection process, we established a screening protocol and used a Quality and Resilience Trigger Tool (please see Aase et al. [12], for detailed information on the screening process and trigger tool). During the selection process we screened a total of 50 research projects (including research projects, post-doctoral projects, and PhD projects) and identified how the projects related to resilience and which quality components they encompassed. As a result, a sample of 25 projects were selected for inclusion to secure a comprehensive range of empirical healthcare settings (e.g., homecare, nursing homes, hospital, prehospital critical care), stakeholders (e.g., family, patients, users, healthcare professionals, managers, regulators), and quality dimensions (patient safety, clinical effectiveness, patient centredness, coordination) [12], see Attachment 1. All researchers in the project agreed upon the inclusion of studies.

### Data collection

From the sample of 25 included projects, we invited 19 researchers to participate in a semi-structured interview. The selection criteria of informants were that the researchers had to be involved in projects that had collected and analysed data, and that had published results, to reflect upon study results and key themes of resilience in healthcare. A total of 16 researchers accepted the invitation, while one rejected it, and two did not respond, making up the data set for this study, please see Attachment 1.

The research team developed a recruitment and interview procedure and a check list based on recommendations for researching researchers [26–29]. This means that when the research team approached colleagues to be recruited to the interviews, we followed a procedure to ensure that all participants were informed and invited in a formal way, they were approached by a colleague who was not in any way in an ongoing or previous power relation to the informant (supervisor, project manager, leader) and the information was sent by email. All participation was voluntary and informed consent was obtained.

The data collection took place between September and December in 2020. All interviews started with an introductory part to explain the RIH programme and why we were interested in their research project and why it had been included in the sample. Moreover, we explained that participants could see the transcripts and approve them if they wanted. The interviews took place face-to-face at a location preferred by the informant or digitally via Zoom and lasted from 75 to 150 min.

The purpose of the interviews was to gain insight into the researchers’ knowledge, experiences and reflections about resilience, adaptive capacity, learning, and the role of stakeholders in resilience as seen from a researcher’s perspective. An interview guide that supported this purpose was developed and used. The interviews were conducted by researchers in the RIH programme, with a varied background including nursing, sociology, innovation, health psychology, and health sciences. The sample included 4 males and 12 females from the age of 30 to 54. We collected data until we reached saturation, where all resilience topics were covered, and a variety of healthcare settings had been included in our data material. Researchers in the sample included PhD students, post doctors, associate professors, and professors.

### Data analysis

All interviews were audio-recorded and transcribed. The data analysis used the NVivo 12 software to structure and provide documentation for the analysis process. The first round of coding followed a deductive pattern guided by the following analytical question:

What type of situations, activities, and practices, illustrating resilient performance can be identified in the data material?

The criteria for coding data as examples of resilient performances were based on Wiig et al. [1] definition of resilience in healthcare: “the capacity to adapt to challenges and changes at different system levels, to maintain high quality care”. As such, coding identified situations, activities, and practices where the outcome revealed the achievement of quality care based on different adaptations to stress and pressure. This first round of coding resulted in 470 instances of resilient performance across different healthcare contexts and levels.

**Fig. 1:**
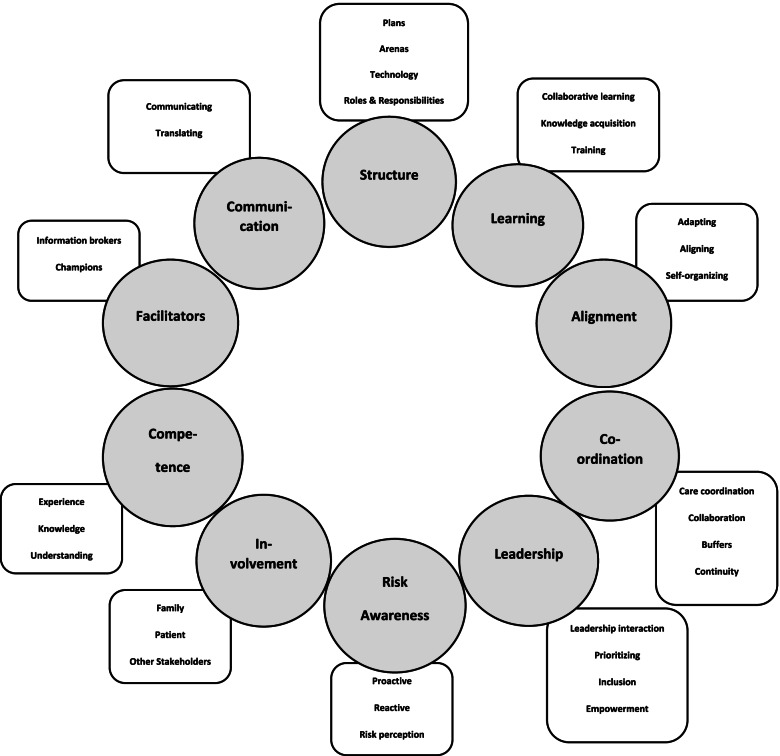
Resilience capacities with associated sub-themes. The capacities within the circles represent 3rd order coding, and the sub-themes within the squares represents 2nd order coding [30]

Having identified instances illustrative of resilience in healthcare, a second round of inductive analysis was initiated in accordance with grounded theory [30, 31]. This second round of coding resulted in 152 different 1st order codes, emerging inductively from the data material. These 1st order codes were aggregated to 2nd order themes and 3rd order dimensions through analytical workshops with the research team (see Fig. 1). The second inductive analysis allowed for codes to emerge directly from the data, thereby grounding the findings in the empirical data. Grounded theory is a methodology found valuable in research seeking operationalization [10].

A key step in, and objective of, the analysis process was to discover capacities for resilience that might be operationalized in future research, and this two-step analytical process allowed the emerging analysis to be aligned with existing concepts and theories of resilience in healthcare. The deductive first round of the analysis made sure that only instances of resilient performance were included for further inductive analysis of facilitative capacities in the second round. This was necessary as the informants described numerous instances with a lack of resilience performance, which if included in the second inductive analysis round could end up misleading. Only material that described occurring or previously occurred resilient performance was included in the dataset, and data material which only described potential actions or resources that could lead to resilience was excluded in this study.

All members of the research team read the data material. Author HBL was responsible for the coding. The results were discussed in several workshops among the research team to ensure consistency and agreement on the themes and the trustworthiness of the process.

**Fig. 2:**
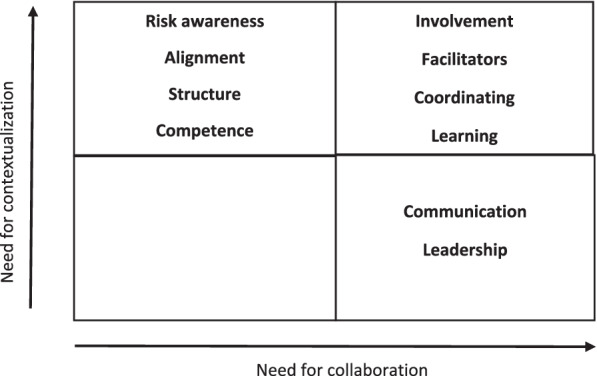
Framework for resilience capacities and their relatedness to contextualization and collaboration

For the development of Fig. 2, the content of all 1st order codes in each capacity were revisited and read over again to identify factors associated with all capacities, based on a thematic analysis framework [32]. From this second round of analysis two themes emerged: contextualization and collaboration. This second analysis was led by HBL and the findings were further discussed among the research team in workshops.

## Results

Based on the data analysis described above, 10 organizational capacities of resilience in healthcare emerged. The 10 different capacities of resilience are presented here in order of those with the most identified instances to those with the least: Structure, Learning, Alignment, Coordination, Leadership, Risk awareness, Involvement, Competence, Facilitators and Communication. Each capacity will be described individually in the following result section. Figure 1 gives an overview over the different capacities (3rd order coding) and their sub-themes (2nd order coding) [30].

### Structure

Structure as a capacity for resilience refers to structures that support work and practise within the organization. Four sub-themes made up the structure capacity: Technology, Roles and responsibilities, Arenas, and Plans, see Fig. 1 and Table 1.

**Table 1:** The distribution of capacity instances, including 2nd order sub-themes, within the dataset

**Structure (244 instances):** Plans (90 instances) Arenas (80 instances) Technology (33 instances) Roles and responsibilities (33 instances)	**Learning (208 instances):** Collaborative learning (74 instances) Knowledge acquisition (67 instances) Training (44 instances)
**Alignment (180 instances):** Adapting (79 instances) Aligning (60 instances) Self-organizing (41 instances)	**Coordination (170 instances):** Care coordination (75 instances) Collaboration (55 instances) Buffers (20 instances) Continuity (20 instances)
**Leadership (157 instances):** Leadership interaction (59 instances) Prioritizing (52 instances) Inclusion (27 instances) Empowerment (19 instances)	**Risk awareness (105 instances):** Proactive responses (57 instances) Reactive responses (22 instances) Risk perception (26 instances)
**Involvement (98 instances):** Family (50 instances) Patients (23 instances) Other Stakeholders (25 instances)	**Competence (84 instances):** Experience (43 instances) Knowledge (31 instances) Understanding (10 instances)
**Facilitators (73 instances):** Knowledge brokers (34 instances) Champions (39 instances)	**Communication (46 instances)** Communicating (33 instances) Translating (13 instances)

Technology involves aspects of accessibility and compatibility of different software and technology. The compatibility between different software systems was emphasized by the informants as crucial to allow for well-functioning information transfers. Another emphasized aspect within this sub-theme was to ensure easy access to information sources, like having access to patient journals on mobile devices.

Roles and responsibilities concerns capacities related to having stability among staff and clearly determined responsibilities both among team members and between different organizations. Stability of staff was experienced by patients and family as valuable in difficult situations and made it easier for them to communicate more openly with healthcare personnel (HCP). Staff stability was also facilitative in terms of treatment structures, for organizing regular meetings, and as support resources. Even though technology was found to be a sub-theme for resilience, the introduction of technology without proper clarification of roles and responsibilities was more of a barrier than a facilitator. For instance, maintenance of telecare tools at patients’ homes was a struggle for homecare services and an extra burden to take on in an already hectic workday. As such, the technology was not perceived as a capacity for resilience unless it was combined with clarified roles and responsibilities.

Meeting arenas were essential to ensure face-to-face communication and learning. Two aspects of these arenas were particularly emphasized by the informants; Having arenas to meet across boundaries, and the presence of arenas for frequent learning. Having frequent and regular learning arenas, e.g., workshops, formal and informal learning arenas, and treatment meetings, allowed for continuous learning and a deeper sense of ownership towards new practices. Furthermore, meeting arenas where HCP could meet across disciplines (e.g., nurses and physicians), across institutions (e.g., HCP from both hospitals and nursing homes/home care services), and arenas that included other stakeholders (e.g., various stakeholder organizations) were emphasized as key for aligning perspectives, interests, and for care coordination. However, informants commonly reported the need for front-line HCP to self-organize to create desired arenas.

Plans occupied a significant part of the Structure capacity and is concerned with plans and procedures of healthcare practices. The integration of quality improvement tools, external quality certifications, and externally driven quality interventions were found to increase HCP awareness of quality and patient safety in care. However, the different improvement methods of choice were of more value when also grounded in management practices. In emergency situations*,* plans were of even more value, due to the need for time-sensitive responses which relied on predetermined roles and responsibilities. For instance, on maternity wards, the women giving birth were closely monitored in terms of potentially adverse situations and if heading towards high-risk situations for the woman or the baby, emergency plans were put into action, with clear responsibilities for all HCP involved.

### Learning

The learning capacity describes how the organization facilitates and provides learning activities and learning opportunities. Learning for resilience was found to include three sub-themes: Collaborative learning, Knowledge acquisition and Training*,* see Fig. 1 and Table 1.

Collaborative learning includes instances where learning was achieved through both formal and informal interactions between HCP (colleagues), with stakeholders (e.g., patients and family), between organizations (hospitals and nursing homes/home care services), and across levels (leaders and front-line staff). Leadership was important for formal learning, either by supporting staff to undertake external courses and education, arranging for inhouse learning opportunities, or by engaging in different improvement projects. Leaders also had an important role in motivating and supporting staff in knowledge acquisition activities and for the translation of new knowledge into practice. Formal learning was found to be an integral part of specialization training for physicians and psychologists. However, nurses reported a lack of formal training opportunities and instead sought new knowledge through informal networks of colleagues and through informal apprenticeship learning from more experienced nurses.

HCP were often initially reluctant to participate in simulation training. Nevertheless, as they started to engage, with some pressure from their leaders, they discovered the value and relevance of simulation training and thus started to appreciate the experience. “Debriefings” with reflections after completing simulation training were highlighted as particularly important.

### Alignment

Alignment referred to various adaptions introduced to bring in line the different external and situational circumstances of what is required at any given time. Alignment as a capacity for resilience includes three sub-themes: Adapting, Aligning, and Self-organizing, see Fig. 1 and Table 1.

Adapting includes adaptations of practices and care to align with specific patient needs. As every patient is unique, care is a complex matter that relies on different types of adaptations to allow for patient centred care. On for instance mental healthcare wards, treatment of suicidal patients needed to extend pre-set safety and treatment measures to provide appropriate care for each specific patient. Adaptations were found to be a way to align the gap between demands (work as imagined) and capacity (work as done).

Aligning includes trade-offs and advocacy to establish shared goals and understanding, which act as a baseline for both efficiency and involvement. This can be exemplified by midwifes who efficiently could develop a shared understanding of birth progression through hand signals, and furthermore, in situations where shared goals between HCP and patients ensured patient involvement in treatment.

HCP were described as a highly adaptive group, where individuals often self-organize to provide high quality care. However, a premise for HCP to be able to self-organize, is leaders who provide HCP the necessary room for manoeuvre for self-organization. Examples of such practices are HCP who searched the internet for information to help patients, or adapted their traditional practices, imported practices and guidelines from external organizations, and even developed their own measures to ensure high quality care, like for instance unofficial checklists.

### Coordination

The Coordination capacity refers to how the organization facilitates and organizes work and further how the organization organizes information flow across different disciplines, levels, and other organizations. Coordination for resilience includes the following sub-themes: Care coordination, Collaboration, Buffers, and Continuity, see Fig. 1 and Table 1.

In terms of care coordination, involvement of the patient’s family was heavily emphasized. The family advocated for coordination of care, influenced decision making concerning their relatives, and acted as knowledge brokers between different institutions (e.g., between hospitals and home care services). Patients not having any family for support was therefore perceived as a risk factor by HCP. Care coordination was also facilitated by specific coordination roles, like the cancer coordinator who followed the cancer patients along their journey and arranged for care across different organizations. As such, this cancer coordinator acted both as a patient coordinator and a buffer for the whole system, taking care of requests from patients which otherwise had to be directed to the physicians or nurses.

Buffer resources were described as a conduit for resilience in healthcare by providing an ability to draw on additional resources in times of need, for instance the arrangement of back-up nurses to call in difficult situations during weekends at nursing homes.

Continuity of staff, resources, and learning was furthermore highlighted as valuable. Leaders with a contextual overview were found to be better at scheduling the different shifts. Having such an overview relied on a familiarity with the staff, which was more easily developed when there was a continuity of both staff and leadership. Continuity of leaders and staff was also found necessary to ensure learning. When healthcare leaders left shortly after completing quality interventions, the achieved learning and improvements gained throughout the intervention were easily forgotten. Furthermore, to optimize practise-based learning, continuity in simulation training (e.g., a short meeting for simulation training every week) was more effective for learning than having less frequent training (e.g., yearly seminars over days and weeks). As such, continuity in training was found to be a facilitator for the development of ownership to new practices, through persistent focus over time.

Inter-organizational and inter-disciplinary collaboration was highlighted as important for shared objectives and for the development of holistic coordination plans. In terms of long-term treatments, like cancer, it was emphasized that patient centred care involves more than having the patient at the centre, it is also to keep a holistic view of the individual. The individual patients have needs, opinions, feelings, and different resources and, as such, different caregivers should collaborate to meet those needs.

Arenas for inter-disciplinary collaboration were reported as something most HCP wanted more of. The increase in digitalisation was found to inhibit tacit knowledge transfer between disciplines, as large amounts of information is now transferred digitally. This can be exemplified by a change in practice at radiology departments. Before the digitalization of images, the radiographers who provided processed images met with radiologists for face-to-face image analysis. These meetings were thus used to function as tacit learning arenas, where knowledge and experiences were transferred across disciplines. With the current digital transfer of images, these meeting arenas have been lost, and with it, the transfer of tacit knowledge between different groups of HCP.

### Leadership

Leadership as a capacity for resilience concerns how leaders facilitate, support, motivate, and contribute to the organization. Four sub-themes made up the leadership capacity: Leadership interaction, Prioritizing, Inclusion and Empowerment*,* see Fig. 1 and Table 1.

In terms of leadership interaction*,* it was found highly important for leaders to provide support and motivation for their staff. For example, leaders arranged for staff to have access to learning arenas and reflexive spaces, sent staff on formal training courses, and they furthermore encouraged empowerment when implementing new practices. Leaders were also key in the development of an inclusive culture, where all employees were allowed to take on responsibilities. However, leaders were also in need of their own support structures, with their being responsible for handling the tension from both the macro and the micro level of the organizational system. Leaders therefore emphasized the importance of networks for healthcare leaders to learn, discuss and ventilate frustration across different organizations.

An important part of leadership is to be able to prioritize between conflicting demands and capacities, and this was reflected in several ways. Firstly, time is an essential factor for leaders when prioritizing. Leaders must decide how much, and for what type of tasks, time could be allotted. Secondly, necessary competence needs to be prioritized for the most demanding and critical situations. Thirdly, leaders must organize resources to ensure both efficiency and needed competency levels, and still operate within the allotted budget. And fourth, leaders need to prioritize in response to the situational risk. All these aspects of prioritization rely on the leader’s contextual understanding and the ability to anticipate, monitor, learn and respond. To handle these tasks, leaders have to be present at the front-line and to take action based on feedback from front-line staff.

### Risk awareness

Risk awareness refers to how the organization understands and reflects on risk that may affect the patient, possible adverse events, and the consequences of actions and adaptations. Risk awareness as a capacity for resilience includes the following sub-themes: Proactive responses, Reactive responses, and Risk perception, see Fig. 1 and Table 1.

Being aware of situational risk is of importance at all healthcare levels to allow for proactive responses. HCP at the front-line cope with uncertainty by interpreting cues and engaging in sensemaking of the available information. Emphasis is given to shared sensemaking in situations of high uncertainty. This can be exemplified by suicide risk assessment on mental health care wards where shared risk assessment was highlighted. Due to the complexity of healthcare, different people often interpreted the patient slightly differently, and bringing these different perspectives to the table was found to allow for a more holistic risk assessment. Adaptations at the front-line were found to result in both successful and unsuccessful situations. Healthcare leaders therefore needed an awareness of the front-line situation to understand the consequences introduced by these adaptations. Furthermore, stakeholders at the macro level must also take risks at the front-line into consideration when forming guidelines and regulations.

Risk awareness often relied on contextual understanding, which means that HCP needed to be responsive to information from different sources, like family and patients, the physical environment and technological equipment. This can be exemplified in the care of terminal cancer patients, where a familiarity with the patient is key. Including different information and perspectives from different sources when forming decisions were described as important in reactive responses.

Communication of risk signals is also a safety issue for organizations that have gaps in competence levels among staff. For example, staff in home care services often include registered nurses, skilled health workers, and assistants. Lack of both clear communication and shared understanding of risk signals were found to be a barrier for risk perception. It was therefore emphasized that healthcare assistants without nursing qualifications need to be trained in different procedures. For instance, they should be trained to always initiate some predefined measures in situations where elderly patients have had a fall. When the healthcare assistants then communicate with nurses at the home care service or with physicians at the emergency department, they can inform them of the results obtained from the predefined measures, thereby having a shared language for communicating risk signals, which eases the risk perception for the nurses and physicians.

### Involvement

Involvement for resilience refers to how the organization introduces and involves different healthcare system actors, and whether the organization systematically gathers information from different sources to obtain a fuller picture of the situation. Involvement was found to include three sub-themes: Family, Patients, and Other stakeholders, see Fig. 1 and Table 1.

Family acted as care givers, care coordinators, advocates, knowledge brokers, and they often influenced decision making among HCP. Even though HCP valued family involvement in caring for patients, systematic approaches for family involvement were often found to be lacking.

Involving patients in their treatment plans was a way of ensuring patient empowerment and ownership, which had a positive impact on patient recovery. An example of patient involvement is where shared decision making between adolescents and HCP at an inpatient mental health ward ensured engagement in the subsequent treatment plan.

Involvement from other stakeholders included that from various interest groups and organizations (e.g., cancer organizations), patients who provided direct feedback of their experiences to HCP for them to improve their practices (e.g., adolescents discharged from inpatient mental health wards), patient liaison resources within municipalities, specialist centres (e.g., cancer centres), and peer navigators. All were found to facilitate important support structures for patients with long-term conditions.

### Competence

Competence for resilience refers to having the appropriate knowledge, attitude, skills, and experience for sound decision-making, being able to take on necessary adaptations, and to have the situational understanding needed to provide quality care. Competence was found to encompass three sub-themes: Experience, Knowledge and Understanding, see Fig. 1 and Table 1.

Experience among HCP is associated with having the necessary contextual overview of their work situation and an ability for easier coordination of daily work practices. As such, it is highly important for HCP in leadership and coordination positions to have an appropriate level of contextual experience. This can be illustrated by an example from maternity wards where highly experienced midwives were assigned to coordinating roles, due to their knowledge and understanding of the context, risk, and processes. In an example from the mental healthcare setting, HCP described situations where they would trust their own professional experience and intuition when evaluating suicidal patients, instead of strictly complying with clinical checklists. Through the development of situated knowledge and experience, for example through simulation-based training, HCP developed ownership of new procedures, facilitating a change in attitude towards their new responsibilities.

### Facilitators

Facilitators for resilience concerns how the organization, or different employees, facilitate for positive impacts for the organization. Two specific types of facilitator roles make up this capacity; Knowledge brokers who facilitate knowledge transfer among colleagues and across boundaries, and Champions who facilitate through their own actions, see Fig. 1 and Table 1.

Knowledge brokers are individuals with familiarity with patients, organizations, technology, or the whole healthcare system. Family is an example of individuals who are knowledgeable of the patient and thereby can act as keeper of the patients’ story. HCP often encourage patients to bring someone familiar with them to the first meeting with the physician or treatment team, to help take note of the information given. Based on the experience of HCP, the patients themselves often felt overwhelmed by all the new information and easily forgot it. In inter-organizational collaborations, for instance between hospitals and nursing homes, knowledge brokers are particularly useful if they are familiar with both contexts. The same holds for knowledge of the healthcare system, where HCP in coordinating roles are perceived more valuable when they have a thorough knowledge of the overall system and, as such, also can act as knowledge brokers within the health system. Super-users of technology and technological systems also act as knowledge brokers who can disseminate their knowledge to their colleagues and be a key representative in communication with the technology supplier.

Champions are individuals who led through actions like motivation, invention, and innovation, and by showing their initiative. Champions are often the first ones to implement new knowledge and practices, which functions as motivation for their colleagues to engage in learning. Champions were also found to act as inventors or innovators. They invented tools and practices if existing solutions were not satisfactory to offer appropriate care. Champions also played a role as initiators where they sought to influence external factors, like by inviting external actors into the team. Champions therefore function as role models who are willing to go the extra mile by taking on additional tasks. This can be exemplified by leaders who step into the role of nurses in peak situations at nursing homes.

### Communication

Communication for resilience in healthcare encompasses the capacity of translating the information to the specific receiver, aligning the message to be communicated to the actual situation, an openness for feedback, and being able to communicate across different actors, levels, and organizations. Translating and Communicating were sub-themes making up this capacity, see Fig. 1 and Table 1.

Communicating includes an awareness of the amount of information to be transferred, type of information, and feedback. In terms of information transfer, it was highlighted that the amount of information given needs to be adapted to the specific patient. Some patients were easily overwhelmed with information, while others wanted all the details. Digital communication tools provided both positive and negative impacts on communication. Compatibility of systems eased information transfer, allowing HCPs to focus communication on tacit elements, like situated experiences, instead of using time in meetings to provide results from clinical measures which could be transferred digitally. However, digitalization was also found to decrease the number of physical meeting arenas and therefore also acted as a barrier for face-to-face communication and tacit knowledge transfer.

Information transfer in the sense of feedback from users/patients/family/other stakeholders was emphasized as valuable. An example comes from an adolescent mental health inpatient ward, where the treatment team asked experienced users to take part in a mock meeting, to help provide feedback of the way in which information was presented to new patients.

To ensure shared understanding across boundaries of experiences, disciplines, organizations and stakeholders, translation of different situated knowledge was often found necessary, e.g., in relation to information transfers between HCP and family. Translation was mostly based on boundary objects, or by involving users and co-researchers with the appropriate contextual knowledge. Boundary objects could be in the form of shared existing frameworks, like for example in one research project that developed a tool where leaders could evaluate themselves on specific quality aspects and agree to further actions for improvement based on how they performed, and the development of local categorizations.

## Discussion

The findings presented above provide an understanding of 10 indicative capacities for resilience in healthcare. Healthcare is known for being highly complex, which is reflected in the number of capacities and furthermore the interdependencies between the 10 resilience capacities. This indicates that the ability of a healthcare system to become resilient is not about achieving success in terms of a single or even just a few capacities but means instead that efforts to uphold a resilient healthcare system are necessarily a consequence of maintaining a holistic awareness of the equal relevance and importance of a wide array of the different resilience capacities identified in this study.

We have attempted to organise the different resilience capacities into a conceptual framework, depicted in Fig. 2. In doing so we found the level of collaboration and contextualization to be purposeful aspects for our understanding of resilience in healthcare. In this framework, contextualization refers to the need for incorporating different context specific aspects (like e.g., experiences, demands, tasks, resources, knowledge, needs) in our understanding of resilience in healthcare, while collaboration refers to the need for engaging and interacting with a diverse range of participants. As such, these terms share some similarities with the “sensitivity to operations” concept from the HRO literature [17, 33], describing interactions and knowledge transfer about situational aspects to create a bigger picture of the context, and where small adaptations may prohibit errors from accumulating.

What stands out in the framework is the empty bottom left box. This emphasises the understanding that all capacities of resilience are related to either collaboration or contextualization or both. Even the content of the bottom right box, communication and leadership, have the potential for being transferred to the top right box. However, as communication and leadership share features with more generic aspects, thereby holding similarities (like face-to-face communication and leaders’ ability for decision making and prioritizing) across disciplines, they have been placed within the bottom right box, even though contextual knowledge may act facilitative in both cases.

Both communication and leadership are described in the literature as important factors in different operationalizations of resilience. Kruk et al. [2], Lee et al. [10] and Barasa et al. [13] all describe leadership to be essential for achieving resilience. In terms of communication, Weick and Sutcliffe [17] describe rapid communication to be a facilitator for resilience, and Barasa et al. [13] state that information management is key for resilience.

If we look at the top left box, we find capacities of resilience highly dependent on contextualization. Risk awareness and Competence, are both important capacities for understanding the impact and outcome of adaptations (which is an important sub-theme within the Alignment capacity) [34]. However, despite the need for contextualization with these capacities, the presence of Risk awareness and Competence does not necessary rely on collaboration (though it is often preferrable), but rather a familiarity of front-line work.

Adaptive capacity has been firmly tied to the resilience concept [1, 2, 10, 13, 17, 34]. Anderson et al. [14] describe adaptations as responses to misalignments between demands and capacity, which are what form work as done. The capacity to maintain high quality clinical practice in situations of high pressure (demands) introduces a need for Adaptations and Alignment to narrow the gap between demands and capacity as illustrated in this study. The uncertainty of complex systems furthermore makes it impossible to foresee all eventualities, leading to a need for Adaptations and Alignment, while the number of responses needed to maintain quality in complex systems are too many to all be included in formal guidelines and procedures. As such, contextual understanding is vital for deciding what type of alignment is needed and what type of outcome the implemented alignments will entail.

Moreover, contextualization was found to be highly important when seeking implementation of new technology, plans, responsibilities, and for the establishment of meeting arenas (all are sub-themes of the Structure capacity). Even though such structural sub-themes can be applied across different organizations, and even industries, these sub-themes need alignment to the target context to be efficient structures. Furthermore, the structural sub-themes all relate to some form of leadership, whether by implementing arenas, plans, distributing responsibilities, or by introducing technology to the organization, illustrating interdependencies between these capacities (Structure and Leadership).

If we move to the top right box, we find capacities for resilience that rely on high levels of both contextualization and collaboration. The Facilitator capacity stresses the need for a high level of contextual competence, like champions who take on extra tasks as a result of their contextual awareness of which resources are needed to manage well in a given situation. Both champions and knowledge brokers perform their role in collaboration with others, either by facilitating shared understanding across different disciplines or stakeholders, or by initiating activities to ensure a positive outcome for the patient, colleagues, or the organization.

The Involvement capacity highlights the value in aligning practice with the individual patient context (e.g., disease, home situation, family situation). Moreover, Involvement naturally relies on a need for collaboration between different actors. Existing literature also emphasizes involvement as important for resilience [35]. Kruk et al. [2] describes involvement of different actors, and Barasa et al. [13] highlights social networks, all pointing to the need for involving patients, family, and other healthcare system stakeholders.

There is a broad consensus in the literature that learning is key for resilience [1, 11, 17, 20]. Within this dataset, Learning to a large extent involved collaborative learning activities, either as apprenticeship learning, practise-based learning, simulation-based training, or in the form of more formal learning arenas. This means that learning is highly related to collaboration and interaction. In terms of patient safety a marked difference of learning in the resilience perspective is to transcend from Safety I, where learning is focused on things that go wrong (adverse events, decrease in quality), to Safety II in which the emphasis is towards learning from things that go well [20].

When knowledge and practices were perceived as valuable for daily work, the motivation to learn increased among HCP, pointing to the need for context specific learning. The Coordination of daily healthcare work was also associated with high levels of contextualization and collaboration. Individuals who possessed a lot of contextual knowledge were often designated to coordinating roles, in which they performed very valuable functions. As such contextualization contributed an increase in the ability to anticipate (knowing what to expect based on contextual understanding) and respond (knowing what to do in that specific situation) when coordinating resources and efforts in care [18].

From the 10 capacities found to provide resilience in this study, there is one specific capacity that stands out from the others, and that is the leadership capacity, which has the potential to influence the rest of the different capacities. Leaders are therefore a critical component for unwrapping the potential of the other capacities, as leaders need to provide the necessary resources for ensuring an effective structure (like technology), learning (like meeting arenas), coordination (like buffers and continuity of staff), room for manoeuvre (allowing for self-organization, alignment and adaptations to take place, and for allowing facilitators to work their magic), distribution of roles and responsibilities (thus providing room for self-organization and facilitators), and ensuring involvement (of external actors, patients and next-kin) [36–38].

In complexity leadership theory, leadership for organizational adaptability in complex organizations is described to call for different leader abilities in order to succeed [39–41]. Leaders providing organizational adaptability need to be generative (facilitating adaptation, autonomy, self-organization), administrative (efficiency, coordination, structure), community-building (involvement and facilitators), information gathering (communication and learning), and information using (risk understanding and competence), hence leadership influences all capacities [39]. However, leadership is not the sole fundament of these capacities, they are all interrelated, and a lack of leadership can be compensated by strong organizational abilities in other capacities.

Our study indicates that efforts to understand or translate resilience capacities into practice need to provide appropriate levels of collaboration and contextualization for intervention activities and for everyday practice. What is clear from our framework is that these translation efforts should involve tailored intervention activities, and material based on this new knowledge about the key role of the collaboration-contextualization dimensions for each resilience capacity. The framework and the inductively arrived resilience capacities constitute a sound basis that will support future resilience learning tools and interventions. The resilience field is a relatively new research tradition in healthcare studies, correspondingly therefore, a translation of resilience in healthcare into healthcare practice is needed. As such, future development of resilience learning tools (such as tools to facilitate reflection) and interventions (such as organizational evaluation tools) can lead the way in supporting organizations in their effort to monitor and strengthen resilient performance [11].

### Strengths and limitations

This study has some strengths and limitations that need to be considered. The study is based on a sample of 16 health services researchers from a Norwegian setting. This form of reflexive research on researchers provides a way of mining the collective wisdom that exist in a community of researchers, but which is rarely explored and mapped systematically. Interviewing colleagues also has major potential strengths as there is key knowledge that may only be held by these informants and which, without systematic exploration, can remain hidden. Collecting information from researchers allows for a more holistic understanding of the field, as the researcher informants can share an overview of their dataset and analysis. Another advantage of using researchers as informants is the possibility to develop a more specified interview guide, as we share a common language and base of knowledge. Having researchers as informants aligns with what Malterud et al. [42] describes as high information power, where all informants were able to provide expert knowledge.

However, reflexive research of researchers also introduces some limitations. The sample could have been larger and included researchers from other Norwegian studies and from international collaborators and projects. Interviewing colleagues has a potential risk of being a limitation if informants who are known to the interviewers think they know more than they actually do, so that the participants can take the information they hold for granted and not share it with the researchers. The development of strict recruitment and interview conduct procedures and checklist were therefore fundamental to ensure a sound research process and the trustworthiness of the results.

We experienced saturation in terms of capacities and settings and a high information richness. Future studies could be conducted with an international sample of researchers, to further validate, support, or revise the framework. The variety of healthcare settings and quality dimensions represented in the sample was considered a strength but could also be a limitation in terms of not reaching enough depth in each quality dimension and resilience topic. The finding during data analysis of an overlapping pattern between the 10 capacities led to the statement of the different capacities being inter-related. However, this interrelatedness has not been studied in-depth, which forms a limitation for this study. Future studies should seek to clarify this pattern of interrelatedness between the different capacities to identify stronger and weaker relationships.

A broad group of researchers were involved in the data collection and analysis. This contributes positively towards the trustworthiness of the results, but also constitutes a potential risk of fragmentation and not seeing the holistic picture. This was mitigated by having a lead researcher (HBL) and a thorough multi-stage process based on consensus between the researchers, from the data collection to the analysis and the writing up of the results.

## Conclusion

The aim of this study was to contribute new knowledge to the discussion of operationalization of resilience in healthcare. This knowledge will be used to develop learning tools for HCP and healthcare leaders. 10 different capacities for resilience in healthcare emerged from the data, which are as follows: Structure, Learning, Alignment, Coordination, Leadership, Risk awareness, Involvement, Competence, Facilitators, and Communication*.* All resilience capacities are interdependent, so effort should not be directed at achieving success by improving just a single capacity but rather at being equally aware of the importance and interrelatedness of all the resilience in healthcare capacities.

A conceptual framework where the 10 different resilience capacities are presented in terms of contextualisation and collaboration was developed. The framework emphasises that all resilience capacities are associated with contextualization, or collaboration, or both, and thereby contributes theorization and guidance for tailor made operationalization efforts for the identified resilience capacities in knowledge translation.

Resilience in healthcare has been found to be a valuable perspective to understand healthcare systems. However, there is a need to ground this perspective among HCP. Future research needs to look at ways in which resilient healthcare can be facilitated and supported in different practice settings, for example through the development of collaborative learning tools. By exploring previous and ongoing healthcare studies, this study provides understanding of different resilience capacities that need to be considered when translating theoretical models to practical improvement in healthcare.

## Supplementary Information

**Additional file 1.**

## Data Availability

The datasets generated and analysed during the current study are not publicly available as the datasets are currently informing other ongoing studies in the Resilience in Healthcare project, but are available from the corresponding author on reasonable request.

## Abbreviations

SHARE: Centre for Resilience in Healthcare
HCP: Healthcare professionals
RiH: Resilience in healthcare
