# Supplementary material for: Capacities for resilience in healthcare; a qualitative study across different healthcare contexts
Source: BMC Health Serv Res. 2022 Apr 10;22:474. doi: 10.1186/s12913-022-07887-6 (PMC8994877; doi:10.1186/s12913-022-07887-6)
Supplement: Supplementary file 1 — Additional file 1. [file 12913_2022_7887_MOESM1_ESM.docx]

|  | **Phenomenon** | **Empirical context** | **Stakeholders involved** |
| --- | --- | --- | --- |
| **#1** | Quality and Safety in hospital care | 10 Hospitals in 5 European countries | Healthcare personnel  Managers |
| **#2** | Shared decision making within metal health | Hospital/Youth psychiatric ward | Youths  Parents  HCP  Healthcare leaders |
| **#3** | Management of quality and patient safety in nursing homes and home care services | Nursing homes and home care services | Managers  Health care professionals |
| **#4** | Hospital admission, hospital discharge, transitional care | Hospitals, nursing homes, and homes care services | Healthcare personnel (ambulance workers, nurses and physicians)  Elderly patients |
| **#5** | Simulation based telecare training in homecare services | Homecare services | Home care professionals |
| **#6** | Transitional care in hospitals, nursing homes, and home care services | Hospitals, nursing homes, and homes care services | Healthcare professionals, elderly patients |
| **#7** | Safe clinical practices for hospitalised suicidal patients | Psychiatric care in a Norwegian hospital | Suicidal patients  Healthcare professionals |
| **#8** | Next-of-kin involvement in cancer care | Cancer ward in two Norwegian hospitals | Next-of-kin  Healthcare professionals  Managers |
| **#9** | Hospital readmissions | The interface between primary (nursing homes) and secondary care (hospitals) | Hospital physicians, nurses, GPs, and nursing home managers |
| **#10** | The use of guidelines in non-traumatic musculoskeletal imaging | Hospitals and general practitioners (GP) | GPs, radiologists, radiological fellows |
| **#11** | Safe use of telecare for elderly patients | Home care services in six municipalities | Home care personnel (nurses and occupational therapists) |
| **#12** | Stakeholder involvement in developing homecare services for senior citizens | Homecare services | HCP  Managers  National authorities  Service users  Next-of-kin |
| **#13** | Medication administration in nursing homes | Nursing homes | Healthcare personnel at nursing homes involved in medication administration |
| **#14** | Supporting care needs of patients with prostate cancer | Person-centred discharge pathway intervention  Hospitals, Home care Services, Cancer support organizations, GPs. | Patients, Healthcare professionals in hospitals (nurses, pathway coordinators, nutritionists, physiotherapists), nursing homes and home care services. GPs. Representatives of cancer support organizations |
| **#15** | Certification for quality in hospitals | Hospitals, certification agency | Hospital healthcare professionals and management  Certification auditors |
| **#16** | Implementation of competence intervention in home care services and nursing homes | Nursing homes  Home care services | Healthcare personnel and managers in nursing homes and home care services |

**Attachment 1. Table displaying included empirical projects from which informants reported**
